# Considering Fish as Recipients of Ecosystem Services Provides a Framework to Formally Link Baseline, Development, and Post-operational Monitoring Programs and Improve Aquatic Impact Assessments for Large Scale Developments

**DOI:** 10.1007/s00267-022-01665-0

**Published:** 2022-05-21

**Authors:** Carolyn J. M. Brown, R. Allen Curry, Michelle A. Gray, Jennifer Lento, Deborah L. MacLatchy, Wendy A. Monk, Scott A. Pavey, André St-Hilaire, Bernhard Wegscheider, Kelly R. Munkittrick

**Affiliations:** 1 Department of Biology, Wilfrid Laurier University, Waterloo, ON Canada; 2 Canadian Rivers Institute, University of New Brunswick, Fredericton, NB Canada; 3 Department of Biology, University of New Brunswick, Fredericton, NB Canada; 4 Faculty of Forestry and Environmental Management, University of New Brunswick, Fredericton, NB Canada; 5 Environment and Climate Change Canada @ Canadian Rivers Institute, Faculty of Forestry and Environmental Management, University of New Brunswick, Fredericton, NB Canada; 6 Department of Biological Sciences and Canadian Rivers Institute, University of New Brunswick, Saint John, NB Canada; 7 Centre Eau Terre Environnement, Institut National de la Recherche Scientifique, Québec, QC Canada; 8 Department of Biological Sciences, University of Calgary, Calgary, AB Canada; 9 Present Address: Institute of Ecology and Evolution and the Wyss Academy for Nature at the University of Bern, Bern, Switzerland; 10 Present Address: Department of Fish Ecology and Evolution, Swiss Federal Institute of Science and Technology (EAWAG), Kastanienbaum, Switzerland

**Keywords:** Ecosystem attributes, Ecosystem services, Environmental impact assessment, Environmental monitoring, Fish, Relevant endpoints

## Abstract

In most countries, major development projects must satisfy an Environmental Impact Assessment (EIA) process that considers positive and negative aspects to determine if it meets environmental standards and appropriately mitigates or offsets negative impacts on the values being considered. The benefits of before-after-control-impact monitoring designs have been widely known for more than 30 years, but most development assessments fail to effectively link pre- and post-development monitoring in a meaningful way. Fish are a common component of EIA evaluation for both socioeconomic and scientific reasons. The Ecosystem Services (ES) concept was developed to describe the ecosystem attributes that benefit humans, and it offers the opportunity to develop a framework for EIA that is centred around the needs of and benefits from fish. Focusing an environmental monitoring framework on the critical needs of fish could serve to better align risk, development, and monitoring assessment processes. We define the ES that fish provide in the context of two common ES frameworks. To allow for linkages between environmental assessment and the ES concept, we describe critical ecosystem functions from a fish perspective to highlight potential monitoring targets that relate to fish abundance, diversity, health, and habitat. Finally, we suggest how this framing of a monitoring process can be used to better align aquatic monitoring programs across pre-development, development, and post-operational monitoring programs.

## Introduction

Prior to commencement of a development project, many countries require an assessment (often called an Environmental Impact Assessment, or EIA) that evaluates the potential impacts of the development (United Nations Environment Programme 2018). The assessment provides information that decision makers can use to evaluate the potential impacts of development against the potential benefits. The EIA is expected to consider both positive and negative aspects of a project and requires justification of recommended decisions with public consultation and scientific opinion (Murray et al. 2018). Generally, these assessments involve predicting impacts from the development based on the current conditions (baseline), project specifications, and available models or analogues. Monitoring is then often required post-development to evaluate if predictions were correct, potentially leading to modification/implementation of mitigation if predictions underestimated or overlooked effects (e.g., Dubé 2003; Kilgour et al. 2007). Development projects include human activities that may have environmental, social, and cultural impacts such as, but not limited to, activities associated with construction of power production infrastructure, mines, mills, or transportation infrastructure.

In any watershed, there are many existing monitoring efforts outside an EIA process, including Environmental Effects Monitoring, regional status and trends monitoring, impact assessment of nearby developments, and project pre-development or baseline monitoring. These other monitoring efforts and the EIA fall under separate regulatory processes, often using different approaches and metrics, resulting in poorly aligned and disjointed evaluation processes (e.g., Dipper 1998). For example, in Canada, there are specific mandatory monitoring protocols for some industries once they are operating (e.g., Environmental Effects Monitoring programs (Walker et al. 2002)), but there is no expectation for monitoring these specific indicators to provide a pre-development baseline, or for making specifical predictions on potential impacts from development (Kilgour et al. 2007). Although before-after-control-impact (BACI) study design was recommended over 30 years ago (Green 1989), the lack of coordination between assessment processes and between monitoring programs means that a BACI approach is seldom incorporated into development assessments (Kilgour et al. 2007; Cronmiller and Noble 2018; Somers et al. 2018). The absence of linkages between monitoring aspects across the different stages of development means that post-development follow-up monitoring programs seldom have an ideal reference database required to effectively detect post-operational impacts or verify EIA predictions. It also limits the monitoring effectiveness for detecting cumulative effects by failing to link the monitoring phases into other regional monitoring efforts.

In many cases the absence of a consistent approach and design for environmental monitoring creates decision-making challenges that have long been a source of frustration (Duinker and Greig 2006; Greig and Duinker 2011, 2014). The design of EIAs needs to consider the focus of follow-up monitoring programs early in the assessment process to allow for the collection of appropriate and adequate pre-development data. The strength of the EIA process is enhanced when there is consistency in evaluation approaches and endpoints between the pre-development (baseline), predictive modeling, and post-operational monitoring using similar, ecologically-relevant metrics to provide meaningful prediction and evaluation of environmental impacts (e.g., Curry et al. 2020). Data collected during each step provides a baseline for comparison and the foundation for modelling to allow the development of testable predictions about post-development environmental conditions. Adaptive feedback loops would improve the effectiveness of development assessments and better integrate the EIA process into regional environmental monitoring and management outside of the development approval process.

The question is “how can we adopt an approach that would better provide opportunities for consistency in monitoring across the phases of development assessment?” Typically, EIAs use a stressor-based risk assessment associated with predicted environmental changes in chemistry, physical stressors, and biomarker responses. The EIA process commonly focuses on valued ecosystem components (VECs) that are chosen at the start of the EIA for ecological significance, social value, or regulatory requirement (Ball et al. 2013). Using stressor-based indicators of VECs assumes adequate understanding of chemical and physical habitat requirements of flora, fauna, and humans, and that the VECs adequately represent the various components of the system (Kilgour et al. 2007). Biological VECs are usually species preferred by society and often do not reflect the most sensitive or responsive elements of an ecosystem or aspects that are easily monitored (Kilgour et al. 2007). Recent EIAs are becoming better at integrating aspects and considerations relevant for the protection of Indigenous rights and practices, and that consider Indigenous knowledge and approaches (Keeyask Hydropower Limited Partnership 2012).

Fish are a common component of EIA evaluations (Ball et al. 2013) because of their relevance for subsistence (including Indigenous harvest), recreational, and commercial fisheries, the assumption that protecting the highest aquatic trophic level will protect the majority of ecosystem levels (Kilgour et al. 2005), and because the abundance, diversity, and health status of fish integrate local environmental conditions (Kilgour et al. 2005), as well as the quality of different habitats used across their life cycle (Schiemer 2000). Pre-development baseline fish data are often restricted to community or biodiversity responses that are difficult to interpret in terms of potential direct effects of the proposed development on fish populations (Munkittrick et al. 2019).

Although integration of better science in EIAs have been suggested for decades, these recommendations are rarely practiced (e.g., MacKinnon et al. 2018). There are a wide variety of approaches to monitoring fish that vary across jurisdictions. Given the socioeconomic and scientific importance of fish and their frequent inclusion in EIA and other aquatic monitoring programs, we will use the example of fish monitoring and assessment in this paper to demonstrate how focusing the aquatic assessment requirements on protecting critical ecosystem attributes to support fish would provide a broad basis for assessment that could provide consistency across the phases of monitoring. This would also integrate and align monitoring associated with the EIA process with other regional monitoring efforts to provide an integrated assessment process. The question becomes what aspects of an environment are critical to be included in such a broad assessment in order to protect fish.

The ES concept (Ehrlich and Ehrlich 1981) was developed during the late-1970s to increase public interest in biodiversity conservation (Gómez-Baggethun et al. 2010) and later refined to create a link between ecological and socioeconomic planning by focusing attention on key aspects and performance measures of benefits that the ecosystem provides for humans (e.g., Millennium Ecosystem Assessment 2005). We think that interpreting that framework from the perspective of the key aspects and performance measures that fish need to thrive would provide meaningful measures of ecosystem health worthy of protection that could be used to define and align key performance measures across the assessment and development process. This paper provides guidance on essential assessment elements during the phases of baseline information collection, development assessment, and post-operational monitoring that would improve continuity, build meaningful linkages, and allow the development of monitoring and management triggers to assist with decision-making. Furthermore, the approach provides a more holistic view of potential impacts.

Although this paper focuses on fish and aquatic environments, the principles presented are applicable to other environmental components. This paper reviews the ES provided by fish, interprets key ecosystem functions from a fish perspective, and finally suggests how the ES concept from a fish perspective could be incorporated into the design of monitoring programs to better align monitoring programs across phases of impact assessment and prediction. Understanding the functions of an ecosystem that are critical for fish will provide the necessary linkages between EIA phases to provide long-term consistency in monitoring needed to improve the ability to protect fish. This discussion focuses largely on freshwater fish components and ecosystem needs, although there would be parallel aspects in marine systems.

## Ecosystem Services Provided by Fish

Ecosystem Services are the benefits that the environment has for human well-being (Millennium Ecosystem Assessment 2005; Suter and Barron 2016). To assist with the organization of ES, there are a few methods, but with little consensus, of categorization (e.g., Boyd and Banzhaf 2007; Wallace 2007; Costanza 2008; Fisher and Turner 2008; Haines-Young and Potschin 2009; La Notte et al. 2017). Here we discuss two common methods for categorizing ES: Millennium Ecosystem Assessment (MA) and generic ecological assessment endpoints (GEAEs). Then, we link them to key ecosystem attributes needed by fish to develop indicators that would provide consistent interpretation across the phases of EIAs. We are interpreting the ES concept consistent with the focus on benefit to human well-being and with the ecosystem aspect providing that benefit as the service (Boyd and Banzhaf 2007; Fisher and Turner 2008; La Notte et al. 2017). It is not the intent to discuss ES provided by fish from an ecosystem accounting perspective, and as such, issues such as “double counting” (Costanza 2008; Fisher and Turner 2008) have not been considered.

The MA was designed to provide decision-makers with an approach to linking aspects of ecosystem assets, services and attributes with human well-being (Millennium Ecosystem Assessment 2005). The MA framework groups the services in four categories: 1) provisioning (the products obtained from ecosystems, including genetic resources, food and fiber, and freshwater, and other similar components); 2) regulating (the benefits obtained from the regulation of ecosystem processes, including aspects such as the regulation of climate, water, and some human diseases); 3) cultural (the nonmaterial benefits people obtain from ecosystems through aspects including spiritual enrichment, cognitive development, reflection, recreation and aesthetic experience, such as knowledge systems, social relations, and appeal); and 4) supporting (the ES that are necessary for the production of all other ES, such as biomass production, production of atmospheric oxygen, soil formation and retention, nutrient cycling, water cycling, provisioning of habitat, etc.). One of the main benefits touted by the MA framework is the integration of ES into policy for effective management towards human well-being. Some credit the MA for the exponential increase in use of ES in decision-making (Gómez-Baggethun et al. 2010).

The United States Environmental Protection Agency (US EPA) developed guidance for GEAEs for environmental risk assessments that use ES (Suter and Barron 2016). The ecological services were divided into seven categories of environmental values (but is not intended to be a definitive or comprehensive list): 1) Consumptive – commodities produced specifically by the environment such as food, timber, and clean water; 2) Informational – natural traits or models for anthropogenic structures, chemicals, and processes such as genetic resources and novel molecules; 3) Functional/Structural – ecological functions and structures such as water regulation, pollination, and nutrient cycling; 4) Recreational – recreational opportunities such as angling, birding, and hiking; 5) Educational – academic and non-academic educational opportunities such as environmental education sites and study areas; 6) Option – environmental preservation for future generations such as delaying a decision when payoffs of irreversible use is unknown; and 7) Existence – non-use without direct service of the environment such as spiritual grounds.

Fish provide a number of direct and indirect benefits for humans that are characterized differently in the MA description of ES and the US EPA GEAEs. To begin to align the concepts embedded within ES with potential assessment indicators in an EIA process, it is necessary to further subdivide the benefits into both the organizational level providing the service (community, population, individual, etc.) and potential endpoints and indicators. Table 1 aligns the direct benefits and two of the key indirect benefits with ES categorization that is likely familiar to practitioners (aligning the MA and GEAE categories). Work completed by Holmlund & Hammer (1999) significantly influenced the ES that we have defined, and below we align them within the four MA ES (provisioning, regulating, cultural, and supporting services) and seven GEAEs (consumptive, informational, functional/structural, recreational, educational, option, and existence). These are discussed by MA and GEAE categories in Supplemental Material 1 to provide the context for aligning ES with potential indicators.

**Table 1:** Key Ecosystem services provided by fish from a human perspective

Service: MA	Service: GEAEs	Benefit	Biological Level	Endpoint	Potential Fish Indicators	References
Direct						
Provisioning	Consumptive	Food	Community/population	Quality and quantity of food (fish)	Abundance, age structure of key species, landings, tissue burden, and tumours and wounds	Chauvin 2018; Food and Agricultural Organization of the United Nations 2020; Holmlund and Hammer 1999; Kuhnlein and Humphries 2017; Miles and Chapman 2006; Modesto et al. 2018; Yep and Zheng 2019
	Informational	Medicinal and other novel molecules	Species	Number and types of compounds available for use	Species richness	Burke 2016; Colwell 2002; Zasloff et al. 2011
		Genetic materials	Community	Genetic resources available for use	Species richness	Noah 2013; Yang and Rannala 2012
		Environmental protection	Individual	Survival, growth, reproduction	None (toxicity test endpoints)	Norberg-King et al. 2018
Regulating	Functional/Structural	Nuisance/pest control	Species	Frequency of disease/parasites	None	Ben-Ami and Heller 2001; Cano-Rocabayera et al. 2020; Noorhosseini and Lakani 2013
Cultural	Recreational	Recreational Fishing and Wildlife Tourism	Individual/population/community	Number of visitor-day opportunities	Fisheries surveys Abundance and age structure of key species	Beardmore et al. 2015; Bessa et al. 2017; Food and Agricultural Organization of the United Nations 2012; Holmlund and Hammer 1999
		Recreational use of water	Ecosystem	Preferred water quality Number of visitor-day opportunities	Balance of piscivores versus planktivores	Schindler 2006
	Educational	Education	Individuals/population/community/ecosystem	Number of visitor-day opportunities	Biodiversity preservation	Clark et al. 2020
	Existence	Cultural significance	Individuals/population/community/ecosystem	Viable population exists	Abundance, age structure	Daily 1997
	Option	Preserve for future generations	Ecosystem	Ecosystem available to future	Biodiversity preservation	Suter and Barron 2016
	Informational	Assessment of ecosystem stress/resilience	Community/population	Changes to community/population over time or US and DS of input	Kills, richness, density, survival, growth, reproduction, condition, anomalies	Holmlund and Hammer 1999; Kelly et al. 2018; Kilgour et al. 2005; Schiemer 2000
Indirect						
Supporting	Functional/Structural	Providing food base for other species of interest	Population	Quality and quantity of food items for species of interest	Abundance, age structure, condition, and tissue burden of prey species	Holmlund and Hammer 1999
		Nutrient cycling	Community	Nutrient levels and origins	Abundance and size structure of key species	Holmlund and Hammer 1999; Schindler et al. 1997; Schindler 2006; Vanni 2002

*MA* millennial ecosystem assessment, *GEAE* generic ecological assessment endpoints, *US* upstream, *DS* downstream

## Ecosystem Attributes Required for Fish to Perform their Ecosystem Services

Fish require a variety of services from the ecosystem to survive. These requirements are reflected in a variety of ecosystem attributes, or characteristics, that can be aligned with provisioning, regulating, and supporting services (Table 2). From a fish’s perspective, the ES are the provisioning services (the products obtained from the environment) of food and the regulating services (benefits from ecosystem processes) of space and habitat for growth, survival, and reproduction. These include adequate environmental conditions for sufficient oxygen, temperature, flow, water quality, and sediment quantity/quality. The support services required by fish include access to adequate habitats and connectivity between habitats. How well the fish performs will depend on the state and variability of these conditions and resources.

**Table 2:** Key ecosystem attributes required by fish for providing a robust environment to enable their ecosystem services

Service	Ecosystem Attribute	Level	Aspect	Environmental Indicators	Fish Indicators
Provisioning	Food	Ecosystem	Food availability and quality	Benthic abundance, primary production, and tissue burden	Growth, reproductive development, condition, tissue burden
Provisioning/Regulating	Dissolved oxygen	Habitat	Oxygen levels	Longitudinal and seasonal oxygen profiles and associated temperatures	Condition, abundance
	Thermal habitat	Spatial-thermal connectivity	Temperature and refugia (area, density)	Longitudinal and seasonal temperature profiles, cold tributaries temperature, groundwater upwellings	Abundance, growth, reproductive development
	Flow	Habitat	Hydrological signatures	Seasonal/daily flow levels/occurrence of channel forming flows, occurrence and duration of low flow events	Condition, spatial distribution
Regulating	Water quality	Habitat	Quality	Water quality threshold exceedances (e.g., pH, turbidity)	Fish health, tissue burden
	Sediment	Habitat	Grain size, distribution, quality	Loads, TSS, substrate composition, chemical contamination	Condition, diversity, tissue burden
Supporting	Spawning, nursery, migratory, and overwintering habitat	Physical structure	Habitat distribution and quality	Key habitat features, recruitment	Size classes, abundance YOY
	Connectivity	Spatial connectivity	Barriers (man-made or natural), flow	Movement, distribution, and identification and characterization of barriers	Genetic diversity, Fixation Index (FST)

*TSS* total suspended solids, *YOY* young-of-the-year

For the purposes of this paper, the ES provided by fish are provided if the fish and their required ecosystem attributes are protected. There is overlap in the characterization of provisioning and regulating services from the fish’s perspective. For example, fish require the provision of sufficient flow, temperature, and oxygen as basics for survival, but their performance can be affected by a failure to regulate these conditions within optimal limits. Within the context of developing indicators that can be used to protect key ecosystem attributes, the characterization of the ES (provisioning or regulating) does not impact the environmental attribute you protect. Examining the ecosystem attributes, and their relevance for ES, is essential for defining endpoints that could be used in each phase. Table 2 summarizes the environmental and fish-specific indicators that are relevant for assessing each ecosystem attribute affecting each of the ES from a fish’s perspective, which are discussed in Supplementary Material 2.

## Monitoring Design Considerations for Incorporating Ecosystem Services and Consistency into Monitoring Programs

A recent review of 339 Environmental Impact Studies found that only 1% used ES and that new methodologies are needed to improve the utility of ES indicators (Sousa et al. 2020). One of the challenges of ES indicators is the absence of clear mechanisms to integrate the data into decision-making processes. We have recently developed a management framework that uses a series of monitoring triggers developed based on pre-development data, forecast triggers developed during the modelling and assessment phases of prediction within an EIA, and management triggers developed from regional planning approaches that effectively links monitoring indicators across phases of monitoring and assessment (Kilgour et al. 2017; Arciszewski et al. 2017; Somers et al. 2018). Developing ES indicators focused on the key ecosystem attributes for fish can provide the necessary linkages between phases to provide long-term consistency in monitoring.

Baseline (pre-development) information on key ecosystem attributes provides information on the current environmental conditions and establishes a recent baseline. Recent baseline data play a couple of key roles in monitoring, including establishing whether current conditions are already altered from historical states (establishing the accumulated environmental state; Somers et al., 2018), establishing a “normal range” to be able to detect post-development change (Kilgour et al. 2017), and helping to focus studies to determine the cause of change (Dubé 2003; Dubé et al. 2013; Kilgour et al. 2007, Arciszewski et al. 2017).

It is important to link the baseline monitoring design to both the developmental assessment and post-development monitoring. Baseline data can be used to inform post-development assessments, and variability in recent data can be used to develop predictions about future expected levels, and feed into models associated with predicting potential development impacts that form an important part of the predictive modeling phase of the development of an EIA. Linking the baseline monitoring design to post-operational assessment will more effectively inform if there are effects from the project. For example, the before-after-control-impact design can eliminate the potential of concluding that there is an effect from the Project when it is actually natural variability (Dubé 2003; Kilgour et al. 2007). In addition, parameters that may be influenced by the project (i.e., fish species that is in future exposure area over lifetime, chemical that is discharged) should be measured.

Having long-term consistency in monitoring provides the opportunity to understand the environmental drivers impacting natural variability in the indicators. Establishing relationships between drivers and responses will inform models to allow predictions of potential development effects (Dubé et al. 2013) and potentially improve model calibration. The predictive modeling ideally provides a forecast based on anticipated environmental conditions (i.e., from the Project, climate change, other projects in area). Being able to make predictions with quantitative indicators based on ecological drivers allows monitoring programs to transition from a retrospective to a predictive focus, with the objective becoming to evaluate how closely the current accumulated state matches the predicted future state from modeling. Given that not all models (either empirical or processed-based) are adequate for extrapolation, model selection and model testing for the anticipated conditions is a required step (Thirel et al. 2015). The model predictions of future environmental performance levels allows the development of “forecast triggers” to be used in adapting monitoring programs (Somers et al. 2018).

Based on the potential changes to the receiving environment, the monitoring plan should be site-specific. Monitoring is the primary mechanism for adaptive management at both project and regional levels. If effects are measured that were not anticipated, the monitoring plan should include mechanisms for adjusting management strategies and mitigation options (Dubé 2003). The post-development monitoring should be focused on monitoring triggers developed from baseline monitoring and forecast triggers developed during EIA modeling activities.

After development, a feedback loop between monitoring and modeling can be used to iteratively improve both activities.

Developing a decision framework for ES for fish components would align monitoring across the development assessment process by improving the consistency of indicators during:

1. Measurement of the current (baseline) conditions during pre-development to site-specifically adapt models for cumulative effects assessment and to establish triggers to adapt to later monitoring phases (monitoring triggers);
2. Modeling the potential impacts of the development based on current conditions and project specifications, and making projections about the potential future state (forecast triggers); and
3. Monitoring consistent indicators post-development to evaluate if predictions were correct and comparing results against both monitoring and forecast triggers (and modifying project and models if predictions underestimated effects; Dubé 2003; Dubé et al. 2013; Kilgour et al. 2007).

Additional triggers available for focusing the monitoring program include performance triggers (set by the engineers to indicate if development is working as designed), compliance triggers (developed from regulatory permits), and management triggers (developed as limits from land use plans or environmental quality criteria; Somers et al., 2018). Exceedance of a management trigger for a stressor indicates habitat quality for biota may be inadequate but this condition may not result in biota exceeding their trigger. Biota exceeding their trigger are more likely to indicate that the overall protection goal is not being met.

The various triggers serve to adapt the monitoring program to increase or decrease intensity of monitoring by indicating when the program should move to a different tier. If over time there are no exceedances of triggers, the monitoring plan may be designed to reduce sampling locations, sampling frequency, and/or indicators measured. The plan should also include triggers that would increase these again if there is a change in indicators (Arciszewski and Munkittrick 2015; Arciszewski et al. 2017).

## Consistency for Aligning Indicators of Ecosystem Attributes for Ecosystem Services with Environmental Assessment Strategies

The previous section highlights the need for tight linkages between the monitoring design, indicator selection, and consistency in data throughout the baseline assessment, predictive modeling, and post-development monitoring phases. The characteristics of ES monitored during baseline should provide the foundations for both predictive modeling and post-development monitoring phases. These linkages will ensure that critical ecosystem functions for fish are protected. The ecosystem attributes required by fish are listed in Table 3 along with the measures that can be taken across the phases of EIA to provide linkages to improve effectiveness, relevance, and consistency and are discussed below. The potential effects of the development should be considered to prioritize the aspects assessed. Using the ecosystem attributes for guidance can shift qualitative statements to meaningful quantitative models and triggers to protect the environment by understanding when there are relevant, project-related changes.

**Table 3:** Measures and uses of ecosystem services from a fish perspective to align monitoring requirements across the phases of environmental assessment

Service	Ecosystem Attribute	Aspect	Assessment Focus	Predictive Modeling Focus	Monitoring Focus
Provisioning	Food	Energy and nutrients	Fish growth, reproductive development, condition, tissue burden Food items biomass, abundance, diversity, tissue burden	Performance levels of key species/food items	Triggers based on normal ranges (baseline variability)
Provisioning/Regulating	Dissolved oxygen	Oxygen levels	Longitudinal and seasonal oxygen profiles	Model critical oxygen levels	Trigger based DO at key times and limiting sites
	Thermal habitat	Temperature and refugia	Longitudinal and seasonal temperature profiles, distribution and area of refugia	Model critical thermal habitat and temperature ranges	Trigger based continuous temperature monitoring at key sites, using remote sensing data to map spatial thermal variability.
	Flow	Ecological flow needs	Targeted ecohydrological variables (flow). Limits of ecological flow alterations.	Model critical flow ranges for target species	Trigger based seasonal/daily water levels, including occurrence and duration of extreme events.
Regulating	Water quality	Ecological water quality needs	Water quality and variation with time and flow	Model water quality	Trigger based for COCs
	Sediment	Ecological sediment conditions	Sediment distribution, grain size, TSS, bedload movement, quality	Model sediment transport and quality	Trigger based at key sites
Supporting	Spawning, nursery, migratory, and overwintering habitat	Ecological habitat needs	Habitat types and quantity and use	Model habitat	Trigger based habitat usage
	Connectivity	Access to critical habitat	Fish movements and critical habitat: Fish tagging studies, tracking studies, presence/absence, size structures, stable isotopes, genetics of populations	Identify critical barriers and issues for movement	Trigger based fish access and passage

*DO* dissolved oxygen, *Temp* temperature, *COC* contaminant of concern, *TSS* total suspended solids

### Measures for Provisioning Services

#### Food

The quality and quantity of food is an essential ecosystem attribute and a pre-development baseline ensures the availability of information for assessing post-development impacts, and for ensuring that modeling during the EIA process focuses on critical aspects. This ES can be measured directly by looking at indicators of food quality and availability, or at certain fish metrics that demonstrate food quality and quantity such as growth, reproductive development, body condition, and tissue burdens of contaminants.

Baseline assessments can focus on direct indicators of food quality and availability, or on indirect fish indicators of food. Direct measures of food quantity and quality can include biomass and taxonomic diversity/composition of food sources (e.g., algae, invertebrates). Bulk biomass estimates of algae through measurement of chlorophyll *a* remains a useful indicator of food quantity at the primary producer level because of the simplicity of sample collection and analysis, and the ease of integration into long-term monitoring processes to assess primary productivity over time. Benthic invertebrate sampling is well established as part of regional and national monitoring strategies (Kilgour et al. 2007; Buss et al. 2015), and there are guidelines in place for study design, sample collection, and sample processing (Canadian Council of Ministers of the Environment 2011; Environment Canada 2012, 2014) that can support their inclusion in EIA. Assessment of invertebrate abundance, biomass, and diversity is commonly used as an indicator of water quality but can also provide information about secondary production and composition that can be used in the context of fish needs as measures of food quantity and quality.

The quality of food is also an aspect to be considered and negative impacts on food quality have been documented. Where potential impacts to primary producers are a concern in the assessment process, the relative proportions of diatoms and other groups such as green algae and cyanobacteria could be used to indicate possible changes to food quality, though fatty acid analysis of algal samples would provide the most accurate measure. For example, eutrophic conditions can result in negative impacts on food quality by shifting primary productivity from predominantly diatoms, which are a high-quality food source for grazers, to an increased dominance of green algae and cyanobacteria, which are lower quality and will affect trophic transfer efficiency across the plant-grazer interface (Goedkoop et al. 2007; Torres-Ruiz et al. 2007). Such a decrease in the nutritional value of primary producers will have implications for growth, hormone regulation, and production at higher trophic levels (Brett and Müller-Navarra 1997).

Fish indicators integrate the critical aspects of food availability, and changes will be reflected in changes in fish performance by alterations in growth, reproductive development, condition, and tissue burdens (Table 3). For example, high availability of food can be reflected at a population level by a lower average age, higher growth rate, higher reproductive output, lower age at maturity, and/or higher body condition (e.g., Gibbons and Munkittrick, 1994; Munkittrick and Dixon, 1989). Since the early 1990s, Canada has had an extensive program for Environmental Effects Monitoring (Walker et al. 2002), and well-documented procedures for assessment of an adult fish survey, including critical effect sizes for interpreting the relative importance of changes (Munkittrick et al. 2009). Guidance for selecting reference and exposure sites, species characteristics, measurement endpoints, and data analysis are available in detailed technical guidance documents (https://www.canada.ca/en/environment-climate-change/services/managing-pollution/environmental-effects-monitoring.html). Additional advice for sampling times (Barrett and Munkittrick 2010) and the development of monitoring triggers from baseline data for these endpoints is available (Kilgour et al. 2017; Arciszewski et al. 2017; Somers et al. 2018).

When sufficient data are available to understand the environmental factors driving natural variability in the baseline endpoints, it is possible to develop correlations between key environmental variables and the measured endpoints (e.g., Kilgour et al., 2019). For temperate freshwater fish, these are commonly related to flow and water temperature, although other abiotic factors could contribute. For lower trophic levels that provide a direct measure of food availability, there may be a stronger relationship with water chemistry and physical habitat conditions such as substrate composition (e.g., Lento et al. 2020). These relationships can be used to provide a bridge between the scenario modeling in cumulative effects assessments for the development conducted under the modeling phase and predicted relevant changes in measurement endpoints. A monitoring plan would include how these triggers are tiered and what follow-up monitoring would be needed to detect post-development impacts.

In an ideal world, post-development monitoring would have both the monitoring triggers from baseline (Somers et al. 2018; adjusted if necessary for more recent data as in Arciszewski and Munkittrick, 2015) and forecast triggers from the modeling phase that would predict the expected normal range of any post-development changes. The monitoring that occurs post-development would monitor the same endpoints of the same fish species and lower trophic levels in the same areas as the baseline assessment (e.g., reference and future exposure). The results post-development would be compared to the triggers that were developed in the monitoring plan. Monitoring would likely occur for multiple years to ensure that any delayed effects are captured and to confirm effects, if differences are seen. If changes are seen that are of concern, monitoring can progress through the tiers of monitoring, to confirmation, extent and magnitude, and if necessary, investigation of cause (Somers et al. 2018).

### Measures for Provisioning/Regulating Services

#### Dissolved oxygen

As a critical ecosystem attribute for fish, adequate oxygenation maximizes growth and carrying capacities (densities), but also determines fish tolerances via the partial pressure difference of fish blood and dissolved oxygen in the water. For most life stages, fish move away from areas with low oxygen and there will be a shift in community to species more tolerant of low oxygen if conditions persist (Tetreault et al. 2013). For those that cannot avoid low oxygen conditions, a small difference, 0.5 mg/L to 1 mg/L, can make the difference between mortality and survival, with lower oxygen level tolerance for shorter exposure periods (Seager et al. 2000). It is generally assumed that eggs are the more sensitive life stage as they depend on a relatively small surface area for respiration (Elshout et al. 2013).

Uncontaminated streams and rivers are generally well oxygenated, so oxygen is not normally a limiting factor for fish in lotic habitats. The current oxygen levels of the water body, including variations over space and time, should be measured before development. Sánchez et al. (2007) found that measuring the dissolved oxygen deficit was sufficient as a rapid indicator of watershed pollution in the absence of a full water quality assessment. Areas that may be susceptible to low oxygen (e.g., stratification, vegetation, groundwater inputs, or zones with elevated biological oxygen demand/chemical oxygen demand) and areas that may serve as refugia should be identified. Future reference and exposure areas should be sampled.

Predictions of dissolved oxygen in the receiving environment after project completion and trigger development are completed during the modeling phase. Oxygen tolerances of fish species can be used to develop triggers from the most sensitive life stage of species present (Elshout et al. 2013). Many jurisdictions already have a water quality guideline for oxygen (e.g., 5.5 mg/L from Canadian Council of Ministers of the Environment (1999)). A follow-up monitoring program with tiers and triggers is developed for post-development.

After the development is complete, the receiving environment should be monitored to confirm predicted oxygen levels. Monitoring oxygen should be similar to what was completed during baseline over space and time and compared to trigger values. The monitoring program moves to the next tier if levels are less than developed triggers.

#### Thermal habitat

Different species have different optimal temperatures (Golovanov 2006) and can be grouped together into coldwater, coolwater, or warmwater thermal guilds (e.g., Coker et al. 2001). Fish generally move at different times of the day and/or seasonally to maintain their preferred temperature. In general, higher water temperatures increases fish metabolism, resulting in increased growth rates as well as greater food and oxygen demand (Todd et al. 2008). Water temperature is an influential ES. Eggs incubating overwinter may hatch in thermal plumes early, resulting in underdeveloped larvae with limited food available (Patrick et al. 2013). Conversely, spring spawning females overwintering in thermal plumes may not develop mature eggs (Golovanov 2013). Fish may be attracted to or avoid warm/cold thermal plumes at different times of the year. Although fish deaths are often thought to be mostly from high temperatures, fish kills occur more frequently from cold temperatures (Beitinger et al. 2000) and can be caused by human activities when thermal discharges abruptly change in effluent flow or water release from a dam (Donaldson et al. 2008).

Before development, the current temperature of the waterbody, including variations over space and time, should be measured (Table 3). This can include in situ measurements and/or remote sensing (i.e., aerial or satellite based; e.g., Dauwalter et al. 2017). Habitats that may serve as refuges during extreme temperatures should be identified and their temporal variability quantified (Dugdale et al. 2013). Future reference (fair thermal conditions) and exposure (potentially adverse conditions) areas should be sampled.

Predictions in thermal plume or changes to the thermal profile from the development are made during the modeling phase. If fish species are known, ideally the optimal growth curve for the most thermally sensitive species and life stages would be used in trigger development (McCullough 2010), but these may not be available for many species. Lethal tolerances and the biological maximum weekly average temperature are not recommended for trigger development for coldwater species (see Donaldson et al. 2008; McCullough 2010). In Canada, common compliance triggers for effluents include a maximum permissible discharge temperature (e.g., 40 °C) and plume Delta T (difference in temperature, e.g., 10 °C). The impact of the proposed development to thermal refuges and access to those areas should also be considered. In addition, mitigation and alternative creation of refuges can be envisaged (Kurylyk et al. 2015). The monitoring plan should include the follow-up monitoring required and tiers the triggers to move from surveillance to possible solutions. Mitigations, such as designing cooling discharges to reduce the adverse changes to the natural thermal regime and biotic interactions or changing flows slowly (Donaldson et al. 2008) can be included. Combining thermal and environmental flow prescriptions should be jointly considered (Olden and Naiman 2010).

If the development is adding a point source thermal plume, monitoring would include a plume delineation (potentially multiple seasonally and/or annually) to confirm the predicted area or volume and temperature (mean, maximum, and temporal variance). Similar temperature monitoring from baseline over space and time is conducted and compared to the trigger values developed in the monitoring plan. If the development has caused an unacceptable change in the thermal habitat (set off a trigger), then the monitoring program moves to the next tier.

#### Flow

Flow and its natural variability are critical for fish assemblage spatial and temporal structure and function in river systems (e.g., Wegscheider et al. 2020); in lake systems analogous measures would be related to depth and waves/currents in shallow lentic habitats (e.g., Duchesne et al. 2021). Quantified by velocity (m/s), water depth (m), and discharge (m^3^/s), variation in flow velocity primarily reflects local-scale measurements directly associated with individual microhabitats while discharge usually represents a reach- or watershed-scale composite variable. Flow is strongly modified by long-term (e.g., climate change, hydropower development) and short-term (e.g., seasonal water withdrawal) flow alterations, for example, through loss of adequate velocity in microhabitats, hydropeaking operations, or changes to long-term discharge patterns, including the timing, frequency, and duration of extreme events (Monk et al. 2011). Changes to velocity and discharge may impact fish condition, for example body shape changes to reduce energy cost in movement (Beachum et al. 2016). The fish community can also change; for example, flashiness or frequent flow fluctuations shifts the fish community to habitat generalists, and reduction in natural flood peaks can increase non-native and flood-intolerant fish species (Poff et al. 2010).

The Ecological Limits of Hydrologic Alteration (ELOHA) framework offers an example of an adaptive watershed-scale framework that considers the human and natural ecosystem for sustainable flow management (Poff et al. 2010). These frameworks can support modeling predictions on how flow will change in response to the development and provide guidance on how flow variation should be quantified, for example moving beyond simple annual and seasonal averages to capture the full ecologically-relevant range of the hydrograph (i.e., magnitude, duration, timing, frequency and variability of flows). Within an EIA, habitats that provide the ecohydrological requirements for present fish species should be identified, including identifying vulnerable connections between different habitats that are required. Triggers should then be based on how flow alteration may influence ecological response. For example, if overbank flow is needed to provide fish access to floodplain habitat, a trigger may be a water level in spring that reaches or exceeds bankfull discharge. If the water level is lower, then further investigation into flood levels and consideration of spring discharge management is considered (Poff et al. 2010). Models can then be developed to support the EIA under different flow scenarios and so velocity, discharge, and bathymetry of the waterbody should be measured prior to development (Table 3). Therefore, an adaptive follow-up monitoring plan with tiers and triggers is needed (Arciszewski et al. 2017; Somers et al. 2018). Monitoring should include measuring daily and seasonal flow, similar to baseline. The flow is compared to the models and triggers developed in the monitoring plan. If there is an unacceptable change in hydrological state of the waterbody after development, then the monitoring program moves to the next tier.

### Measures for Regulating Services

#### Water quality

Water quality is important for many aspects of fish health and function including osmoregulation, clarity for vision, and exposure to toxic substances. While water quality is relatively easy to assess at a single location and time, it can be a challenge to assess as fish are often moving within a system and can be exposed to a variety of waters of various quality over time. Water quality will also vary among regions, within a watershed, and temporally (i.e., diurnal, seasons, and years). An effective metric will consider all of these characteristics beginning with what the fishes require (e.g., Canadian Council of Ministers of the Environment), current conditions and trends, and assessing a parameter that may be altered over time, e.g., during a development project. Many water quality parameters react or bind with other components in an aqueous environment, which can influence toxicity. Therefore, measuring these other influencing factors, such as hardness (calcium and magnesium), dissolved organic carbon, pH, temperature, alkalinity, and sulphate (Paquin et al. 2002) are important to consider.

Some water quality parameters vary with flow, and some seasonally, making the development of monitoring triggers more challenging. Based on the project description and baseline data, changes to water quality parameters are predicted in the modeling phase and to develop forecast triggers. If sufficient baseline data exists, monitoring triggers can be developed based on normal ranges, control charts, or other methods (Burgman 2005; Arciszewski et al. 2018). There are toxicity data available for many chemical parameters for many fish species and life stages and should be considered in the development of management triggers. Many jurisdictions have water quality criteria that are influenced by toxicity data (e.g., Canadian Council of Ministers of the Environment). A follow-up monitoring plan with tiers and triggers for an adaptive program should be developed.

If the development is adding a point source, monitoring would include a plume delineation (potentially multiple seasonally and/or annually) to confirm the predicted size and concentrations of parameters. Similar water quality monitoring to what occurred during baseline is conducted and compared to the trigger values developed. If the development has caused a parameter to exceed a trigger, then the monitoring program moves to the next tier.

#### Sediment

Sediment impacts habitat quality in a variety of ways, including impacts on water quality, substrate quality, and as a mechanism of contaminant transport. Suspended sediment also reduces water clarity, affecting predator-prey effectiveness and other stresses (e.g., Ortega et al. 2020), and many species prefer specific grain sizes during different life stages and sudden changes, such as deposition over eggs, can be harmful to survival. Sediment quality may be a source of contaminants for fish (Affandi and Ishak 2019).

Before the development project starts the sediment characteristics that the development would potentially influence, such as grain size distribution, bedload movement, suspended solids, and/or sediment quality should be evaluated. There are multiple sources of sediments in a watershed, some of which are on the drainage basin, while others are in-stream. The relative importance of these various sources to river sediment loads may shift with changes in land use and/or river hydrology and hydraulics (Liu et al. 2018). Although sediment may not be directly influenced, changes to water quality or flow from the project may change sediment quality, distribution, or movement. Based on species present, habitat that is chosen largely based on sediment, such as spawning grounds, should be identified. Changes may occur in sediment distribution naturally with changes to water flow and load, and seasonal measurements over multiple years will help to understand this variability (Zhou et al. 2018). Future reference and exposure areas should be sampled.

During the modeling phase, the effect of the development on sediment is predicted. Triggers are developed that can be based on the normal ranges in quality and quantity, toxicity, and/or predictions of effects. These triggers are then tiered in a monitoring program. In Canada, for instance, guidelines and triggers are specified for different water uses (Canadian Council of Ministers of the Environment, https://www.ccme.ca/en/resources/water/index.html). Those relating to the protection of aquatic life allow for an absolute (or sometimes relative) exceedance of the regional average. Thus, a regional analysis is required to implement such recommendations. This can be achieved through antecedent (pre-project) monitoring, or by using modeling tools (e.g., Sirabahenda et al. 2017).

Monitoring should measure many of the same aspects that were measured during baseline to determine if predictions were correct. If the development has caused an unacceptable change in sediment, then the monitoring program moves to the next tier.

### Measures for Supporting Services

#### Spawning, nursery, migratory, and overwintering habitat

Most fish have different habitat requirements for reproduction, different life stages, and/or seasonally and fish require access to these habitats at the appropriate time for survival. Before development the amount and types of habitat upstream and downstream of the development should be measured and the use of these habitats by fish species should be determined. There are various methods for collecting habitat use data, including biotelemetry (e.g., Capra et al. 2017), direct observation, and direct capture of individuals across different habitat types (Spurgeon et al. 2019; Wegscheider et al. 2020). Quantifying the location, size, and quality of these crucial habitats, through the means of field-based surveys or remote sensing techniques (i.e., aerial or satellite based multispectral imagery; Hugue et al. 2016; O’Sullivan et al. 2020), represents an important ingredient for a large-scale environmental assessment. However, the availability of suitable habitat varies with flow, which results in spatiotemporal patterns of habitat availability and utilization (Wolter et al. 2016).

Habitat use data can be collated and expressed using habitat preference functions using a variety of statistical methods (Ahmadi-Nedushan et al. 2006), which result in a habitat suitability index – typically ranging from 0 to 1 – and can be supplemented with expert knowledge when availability of habitat use data for several species is limited (Mocq et al. 2013; Wegscheider et al. 2021). When the critical habitats in the vicinity of the project area are identified then the project description can be used to predict how habitat suitability may change over time and in response to environmental impacts. Triggers can be developed for habitat utilization as well as quantity and quality of critical habitat.

Predictions can be validated by monitoring habitat use of target fish species, assuming that target species would favour highly suitable habitat over areas predicted to be of poor quality, and monitoring data compared to triggers developed for the monitoring program. The monitoring program moves to the next tier if triggers are set off.

Despite its benefits, there are several limitations of physical habitat as a diagnostic tool in EIAs. First, habitat availability does not represent habitat use, which means that the presence of suitable habitat conditions does not necessarily result in the presence of the species of interest. Furthermore, especially in the case of EIAs, the biological response or recovery after an impact might lag the direct response of abiotic factors, which could potentially lead to erroneous conclusions.

#### Connectivity

As discussed in previous sections, fish need different habitats at different life stages as well as access to refugia during environmental extremes. These areas need to be connected, by passable waters, for fish to survive (Schiemer 2000; Magoulick and Kobza 2003). In addition, populations of fish have degrees of connection between them, which determines the spatial scale for genetic diversity. Barriers to fish connectivity can have negative consequences for effective population size (Gouskov et al. 2016). Where significant barriers will be created or when endangered, rare, or threatened species are present, more intensive sampling (i.e., genetic) should be considered. The species present, their required habitats during different life stages and seasons, and current status or future status of connectivity needs to be understood for this ES to be adequate.

Prior to development, where populations of present fish species are and how they interact may be important to understanding the full potential impact of the development. The distribution of species can be assessed with traditional sampling approaches (nets, electrofishing), but can be labor intensive. More recently developed approaches have used genetic barcoding, either through tissue sampling (can occur starting with a non-lethal fin clip) or Environmental DNA (eDNA) filtered from water samples. Genetic analysis can take a one-species-at-a-time approach with species specific probes and primers in a Quantitation Polymerase Chain Reaction (qPCR), or a metabarcoding approach with PCR amplification of the barcode region followed by amplicon Next Generation Sequencing (Ivanova et al. 2007). The former approach has the advantage of higher precision and accuracy, but will miss species not specifically tested (including species unknown to science). The latter approach is less accurate but does not require a priori knowledge of species diversity.

Observations through tagging and acoustics, or stable isotope analysis can provide information on movement of species of interest and the presence of critical barriers that could limit connectivity between habitats. Genetic analysis can provide information of diversity at multiple levels (individual, population, metapopulation, glacial lineage, and species levels). Population genetics for each species can be determined in a variety of ways. Genotyping microsatellites provides excellent information on a within-catchment scale (Selkoe and Toonen 2006), but they must be developed for each species independently. Microsatellites are highly variable repeated stretches of DNA that provide valuable information about neutral genetic variability that can be informative to infer effective population size, inbreeding, and population connectivity. Restriction-site-associated DNA tag markers (RAD-seq) can be generated without species-specific a priori tool development (Miller et al. 2007). They can provide all of the neutral patterns as microsatellites, and additionally provide information on adaptation. However lab work and bioinformatics for RAD-seq is substantial and expensive. Baseline data can be used to develop monitoring triggers for numbers of fish that do not have passage and access.

If the project is adding a potential barrier of connectivity between populations or required habitat, the modeling phase should predict how it will affect the fish species present. After development, monitoring could employ some of the baseline but also additional genetic approaches, depending on the relative level of impact predicted during the modeling phase. Traditional fish sampling approaches and/or eDNA with species-specific primers and probes can be used to monitor presence/absence distribution changes. With some species, semi-quantitative abundance estimates may also be possible (Wood et al. 2021). The population genetic analysis described for the baseline phase above are not useful for immediate post-development monitoring, as the patterns reflect demographic changes occurring on decadal and longer timescales (Jordan et al. 2013). Instead, the same genetic data can be used with individual-level analyses as opposed to population-level analysis. For example, genetic mark-recapture and parentage analyses can measure migration occurring year-to-year as well as census population size (Miller et al. 2005). Transcriptomic analysis (measuring levels of gene expression) of unhealthy fish could provide information on the physiological cause of the problem (e.g., metal toxicity, heat shock, etc.). If the distribution of species is changing, fewer fish are moving between areas than predicted, or abundance of populations are declining, the monitoring program would move to the next tier. The post-development monitoring program would include tiered triggers to assess the site-specific impacts of the development.

## Conclusions

The EIA process would be improved by developing consistency in monitoring approaches conducted prior to, during, and following the completion of an EIA. Baseline monitoring and modelling need to be aligned so that they support post-development assessment. Interpreting the key ecosystem attributes required for fish to thrive provides one example of an approach that can be used to provide consistency in indicators and to develop that foundation for post-development evaluation. It is critical that post-development monitoring requirements are considered early in the development of the EIA. Pre-development monitoring need to focus on the development of the baseline necessary for identifying the significance of any changes post-development and focuses modelling approaches so that predictions of the magnitude of expected change can be pre-defined so that EIA predictions can be assessed.

Interpreting the key ecosystem attributes through a lens of Ecosystem Services that fish require defines measurable attributes that will form key components of post-development evaluation. It also defines clear objectives for baseline monitoring (the development of monitoring triggers or thresholds that will define change) and focuses modelling to define quantitative predictions (forecasts) of the level of expected change (or absence of) that provide the foundation for the post-development assessment process. The post-development monitoring plan can include mechanisms for adjusting sampling and management by using triggers to move between stages, or tiers, of the plan. These changes can shift current EIAs from qualitative statements to meaningful quantitative models and triggers that will protect the environment by understanding when there are relevant, project-related changes. The EIA process needs to provide the information on which management decisions can be made. Utilizing consistent indicators across the development process and aligning them with other monitoring conducted within a basin offers the opportunities to use a before-after design and improve the capability of determining if changes seen post-development are caused by the project and provides a broader regional perspective and capacity for integration into regional management approaches.

## Supplimentary Information

SUPPLIMENTARY INFORMATION
