# Supplementary material for: Considering Fish as Recipients of Ecosystem Services Provides a Framework to Formally Link Baseline, Development, and Post-operational Monitoring Programs and Improve Aquatic Impact Assessments for Large Scale Developments
Source: Environ Manage. 2022 May 21;70(2):350–67. doi: 10.1007/s00267-022-01665-0 (PMC9252955; doi:10.1007/s00267-022-01665-0)
Supplement: Supplementary file 1 — SUPPLIMENTARY INFORMATION [file 267_2022_1665_MOESM1_ESM.docx]

# SUPPLIMENTARY INFORMATION

1. ECOSYSTEM SERVICES PROVIDED BY FISH

These sections provide more discussion for **Table 1**.

- 1. Provisioning (MA Category) / Consumptive/Informational (GEAE Category) Services

Fish provide food for humans both directly, with 156.4 million tonnes (including finfish, shellfish, and other aquatic invertebrates) consumed in 2018 worldwide (Food and Agricultural Organization of the United Nations 2020), and indirectly by providing livestock feed and crop fertilizer from fish meal and waste (Miles and Chapman 2006; Fréon et al. 2014; Chauvin 2018; Yep and Zheng 2019). An additional way fish provide food indirectly is by acting as hosts for freshwater mussels (Modesto et al. 2018), which can be a traditional food item of Indigenous peoples (Kuhnlein and Humphries 2017). The quantity of fish available as food has been impacted by overfishing (Food and Agricultural Organization of the United Nations 2020) and quality of fish as food has been impacted by contamination (e.g., mercury Government of Canada 2016) for some species in some areas.

Fish provide value for humans in other manners including the provisioning of medicinal products (Zasloff et al. 2011), as a source of materials for medical and pharmaceutical research (e.g., tetraotoxin; Colwell, 2002), for improving agricultural production through genetic selection (Noah 2013), for studying phylogenetics to interpret species relationships and evolutionary patterns (Yang and Rannala 2012), as a model vertebrate in medical and other research (Burke 2016), and as toxicity testing organisms for regulatory purposes (Norberg-King et al. 2018). There are molecules and genes within fish that we do not know of or understand, so preserving biodiversity will ensure continued existence of these unidentified molecules for future study and discovery (e.g., Baumgärtner 2007).

- 1. Regulating (MA) / Functional/Structural (GEAE) Services

Fish function as a biocontrol of pests, including control of mosquitoes (Cano-Rocabayera et al. 2020), snails (Ben-Ami and Heller 2001; Ledford and Kelly 2006), and filamentous algae, floating weeds, and rice pests (Noorhosseini and Lakani 2013), thus contributing to disease prevention and protection of habitat. Fish can also be important in control of primary production which can otherwise lead to algal blooms (Bernes et al. 2015).

- 1. Cultural (MA) / Recreational, Educational, Existence, Option, or Informational (GEAE) Services

Broadly, fish provide value in terms of socio-economics, i.e., providing income while supporting cultural practises (Food and Agricultural Organization of the United Nations 2012). Recreational fishing similarly provides economic support (Carlén et al. 2021) and social value (Beardmore et al. 2015), as does dive tourism (Bessa et al. 2017; Tribot et al. 2018). Similarly, the food base that fish provide for many birds and mammals service popular wildlife and ecotourism industries (Holmlund and Hammer 1999). There are also widespread goals to increase the general public’s environmental literacy and improve conservation outcomes (Clark et al. 2020), including use of fish as the centrepiece of educational efforts such as aquariums and fluvariums.

Cultural components can be grouped conceptually with GEAE informational values such as existence, option, and informational services. Existence benefits are associated with cultural satisfaction that species are present (Daily 1997). Option benefits are associated with the preservation of potential uses for future generations, such as delaying resource decisions on a precautionary basis (Suter and Barron 2016). Informational services are associated with embedded information on ecosystem health such as stress (Schiemer 2000; Kilgour et al. 2005) and resilience (Holmlund and Hammer 1999; Kelly et al. 2018).

- 1. Indirect Supporting (MA) / Functional/Structural (GEAE) Services

Fish provide a wide variety of supporting services that benefit humans. The ones that are most relevant for the purposes of this paper are their roles as a keystone species that provides a food base for other species of interest (Holmlund and Hammer 1999), their role in nutrient redistribution between habitats and ecosystems (Vanni 2002), and their influence on structure and productivity of food webs (Schindler et al. 1997; Schindler 2006).

1. ECOSYSTEM ATTRIBUTES REQUIRED FOR FISH TO PERFORM THEIR ECOCSYTEM SERVICES

These sections provide more discussion for **Table 2**.

- 1. Provisioning and Regulating Services

The quality, quantity, and distribution of food resources is key for fish survival. Food quality and availability may be useful as a direct indicator of ES the fish require, and the direct measure of growth, survival, reproduction, and body condition of the fish as an indicator of this ES performance (Munkittrick and Dixon 1989; Gibbons and Munkittrick 1994; Chase et al. 2016). Fish will also be affected by competition for food and can show responses in terms of population size (Andersen et al. 2017) and community structure (Sandlund et al. 2013). It is, however, not always a simple link between food resources and health of fish populations. For example, fish health can be impacted by contaminants (e.g., van der Oost et al. 2003) as well as disease and parasites (Bricknell et al. 2006; Barber and Scharsack 2009). Regardless of how direct the linkage is between food resources and fish performance; food resources are an essential attribute to protect. Fish food (i.e., plankton, benthic invertebrates, other fish) and fish populations can be measured as indicators of whether the provisioning service of food is adequate. Impacts to these endpoints can be affected by a variety of other ecosystem attributes and defining the cause of changes can be difficult but linking indicators of quality across the phases of environmental assessment is what we are advocating for.

Dissolved oxygen, temperature, and flow are both provisioning and regulating services, as fish need them for survival, but failure to regulate these conditions within the limits required by the fish will affect the ability of fish to survive. Provisioning services are components provided by the environment, whereas regulating services are the benefits provided by ecosystem processes.

Dissolved oxygen content varies within or between water bodies, due to water temperature (higher temperature, lower oxygen solubility), biological and chemical oxygen demand (respiration, decomposition, oxidation), primary productivity (produce oxygen during day), water surface-atmosphere interface (ice cover, total surface area to volume), and groundwater inputs (potentially low oxygen; Canadian Council of Ministers of the Environment, 1999). Although there are some fish species adapted for low oxygen levels or that have some capacity to take oxygen from air, most species have limited activity, function, and survival in increasingly low oxygen situations (Currie and Evans 2021). Understanding seasonal profiles and critical oxygen requirements is important for development of appropriate metrics to evaluate the dissolved oxygen attribute.

Most fish species are ectotherms (Golovanov 2006) and their body temperature is 0.1 to 1^o^C warmer than surrounding water temperatures (Beitinger et al. 2000). Water temperature changes diurnally and seasonally and can vary vertically and horizontally within a waterbody, thus influencing fish body temperature. Each species and each of their life history stages will have an optimal temperature range, and each population can adapt to somewhat from that range depending on the environmental conditions (e.g., Eliason et al., 2011). In most cases where fish live within their optimal temperature range, a warmer environment will tend to increase somatic growth (Dhillon and Fox 2004) relative to colder environments. This difference can be reflected in growth rate and thus impact age at and rate of maturity (Neuheimer and Grønkjær 2012). Understanding the seasonal profiles of mean temperature, its variance at different temporal scales (e.g., daily, seasonally), and the preferred range and critical temperatures of fish species is necessary to understand thermal habitat attributes.

Other aspects of the physical environment also provide and regulate the state of health of a fish. Variability in depth and flow over time as water moves through the wetland, lentic and lotic, and estuarine components of a river network influences habitat quantity, quality, and connectivity. For example, variability in river flow can be a strong determinant of aquatic community structure and function affecting species abundance, total biomass, and diversity via direct (e.g., habitat availability and connectivity, thermal regimes, or sediment movement) and indirect pathways (e.g., water quality, food supply, habitat suitability; Jaeger et al., 2014; Jellyman et al., 2013; Poff et al., 1997; Ríos-Pulgarín et al., 2016; Wegscheider et al., 2020). While fish are adapted to the dynamic nature of most flowing water environments, an alteration of flow, e.g., dams or water withdrawal, can be presumed to alter the fish health (Torralva et al. 1997; Benejam et al. 2016) and community structure (Bunn and Arthington 2002; Beachum et al. 2016; Macnaughton et al. 2016; Patrick and Yuan 2017). An understanding of flow regime is crucial for understanding the flow attribute that fish require from their environment.

Physical environments are also important in lake environments. Lentic or standing water environments can have dynamic shorelines and develop currents based on discharges (runoff, streams, and groundwater), wind, density (influenced by temperature, ice, salinity, and particle and gas concentrations), and bathymetry (McGinnis and Wuest 2005). A variety of habitats can develop from these variations which fish use for rearing, feeding, and escaping competition and predation (Stoll et al. 2008; Bertolo et al. 2011; Callaghan et al. 2016). Understanding the seasonal and daily profiles of flow and water levels as well as the requirements of fish species is necessary to the development of appropriate metrics to assess flow attributes.

- 1. Regulating Services

Water quality and sediment need to be regulated within limits for fish survival. Water quality can vary greatly between and within waterbodies. Each fish species (and population) is adapted to a specific range of concentrations for specific ions such as sodium, calcium, and chloride to maintain osmoregularity (Evans et al. 2005; Hwang et al. 2011) and have varying tolerance to other chemicals, parasites, and pathogens in the water column. Inadequate water quality can be associated with a variety of sublethal effects or death. Toxicity is generally evident with increased liver weight and decreased condition and gonad weight or metabolic disruption (Munkittrick et al. 1991; Hewitt and Servos 2001), but contaminants can also interfere with lateral line or olfactory function, reducing fitness and survival (Tierney et al. 2010; Mogdans 2019). Indirectly, water quality can influence food availability (Robinson et al. 2016) and dissolved oxygen concentrations (Sánchez et al. 2007) which may lead to a shift in the fish community composition (Brown et al. 2011) or fish health (Munkittrick and Dixon 1989; Gibbons and Munkittrick 1994). The fluctuations of key water quality parameters, as well as performance thresholds for fish species will be key attributes to consider during monitoring and assessment.

Sediment (both suspended and bed) effects on fish can vary between and within waterbodies (e.g., Berry et al. 2003). Sediment can impact fish either directly by impacting gill function (Lowe et al. 2015), burial of embryos (Sternecker and Geist 2010), or decreasing visual acuity (Kjelland et al. 2015) or indirectly by altering food availability or introducing contaminants (Bilotta and Brazier 2008). Understanding the fluctuations of sediment load, its composition and quality, as well as the habitat requirements of and chemical threshold for fish species are important metrics to assess fish and this attribute.

- 1. Supporting Services

Supporting services are necessary for fish survival and for the production of other ES, and include oxygen production, nutrient cycling, and water cycling. Direct measures of these ES are not often a primary component of a monitoring program as these processes are reflected in productivity and performance of the system. Monitoring of these aspects would be triggered when there is a dysfunction in the system and more information about the cause of the dysfunction is needed. In terms of fish, critical habitats and connectivity between those habitats are the primary supporting attributes fish require to be successful.

Fish habitat requirements can vary seasonally, with the reproductive cycle, life stage, and between species. Spawning, rearing, feeding, and overwintering habitats can be very different. Connectivity between these habitats is essential for fish. Habitats must be maintained during critical life stages, such as egg incubation, or change subtly to accommodate rapidly changing body size and energy needs, such as required for fry and juveniles (Schiemer 2000). Many species require different habitats between spawning, nursery, juvenile, and adult life stages, and the movement of fish between habitats may be affected by movement barriers such as dams. The most obvious fish affected by barriers are diadromous fish, which migrate between fresh and saltwater. Extreme habitat conditions also occur periodically, such as low flows, low oxygen, and high temperatures in summer. Understanding key habitat features for species present, including refugia habitat is crucial for survival (Schiemer 2000; Magoulick and Kobza 2003).

Populations of fish (groups of interbreeding individuals), do not exist in isolation, but rather with degrees of connection through migration to other populations in different reaches, tributaries, rivers and watersheds (Allendorf et al. 2012). The spatial scale of a single population can vary widely, with the American Eel (*Anguilla rostrata*) having only a single population in the entire species, which spans rivers from Cuba to Iceland (Côté et al. 2013), while Brook Trout (*Salvelinus fontinalis*) populations can be restricted to a single reach of a river. The effective population size may also be important to understand, as it can be influenced by stochastic factors (e.g., a “bad year” that few individuals survived, year of unequal sex ratio) and life history (e.g., mating system where few dominant males fertilize most females; Frankham, 1995). Effective population size is inversely proportional to random changes and loss of genetic diversity each generation, known as genetic drift (Frankham et al. 2002). It is important to understand how populations are distributed within and among waterbodies, because this determines the spatial scale for genetic diversity assessment and management programs. Barriers to fish passage include dams, culverts, and waterfalls, but can also vary temporally due to stream flow variation (Bourne et al. 2011). Given enough time, dams and other barriers to fish connectivity can have negative consequences for effective population size (Gouskov et al. 2016). Understanding critical habitats and connectivity between those habitats is important for development of appropriate metrics to evaluate these supporting ES.

1. Supplementary Material References

Allendorf FW, Luikart GH, Aitken SN (2012) Conservation and the Genetics of Populations, 2nd edn. Wiley-Blackwell, Chichester, United Kingdom

Andersen KH, Jacobsen NS, Jansen T, Beyer JE (2017) When in life does density dependence occur in fish populations? Fish Fish 18:656–667. https://doi.org/10.1111/faf.12195

Barber I, Scharsack JP (2009) The Three-spined Stickleback – *Schistocephalus solidus* system: An experimental model for investigating host-parasite interactions in fish. Parasitology 137:411–424. https://doi.org/10.1017/S0031182009991466

Baumgärtner S (2007) The insurance value of biodiversity in the provision of ecosystem services. Nat Resour Model 20:87–127. https://doi.org/10.1111/j.1939-7445.2007.tb00202.x

Beachum CE, Michel MJ, Knouft JH (2016) Differential responses of body shape to local and reach scale stream flow in two freshwater fish species. Ecol Freshw Fish 25:446–454. https://doi.org/10.1111/eff.12225

Beardmore B, Hunt LM, Haider W, et al (2015) Effectively managing angler satisfaction in recreational fisheries requires understanding the fish species and the anglers. Can J Fish Aquat Sci 72:500–513. https://doi.org/10.1139/cjfas-2014-0177

Beitinger TL, Bennett WA, McCauley RW (2000) Temperature tolerances of North American freshwater fishes exposed to dynamic changes in temperature. Environ Biol Fishes 58:237–275. https://doi.org/10.1023/A:1007676325825

Ben-Ami F, Heller J (2001) Biological control of aquatic pest snails by the Black Carp *Mylopharyngodon piceus*. Biol Control 22:131–138. https://doi.org/10.1006/bcon.2001.0967

Benejam L, Saura-Mas S, Bardina M, et al (2016) Ecological impacts of small hydropower plants on headwater stream fish: From individual to community effects. Ecol Freshw Fish 25:295–306. https://doi.org/10.1111/eff.12210

Bernes C, Carpenter SR, Gårdmark A, et al (2015) What is the influence of a reduction of planktivorous and benthivorous fish on water quality in temperate eutrophic lakes? A systematic review. Environ Evid 4:1–28. https://doi.org/10.1186/s13750-015-0032-9

Berry W, Rubinstein N, Melzian B, Hill B (2003) The Biological Effects of Suspended and Bedded Sediment (SABS) in Aquatic Systems: A Review. National Health and Environmental Effects Laboratory, United States Environmental Protection Agency, Rhode Island, USA

Bertolo A, Pépino M, Adams J, Magnan P (2011) Behavioural thermoregulatory tactics in lacustrine brook charr, *Salvelinus fontinalis*. PLoS One 6:18603. https://doi.org/10.1371/journal.pone.0018603

Bessa E, Silva F, Sabino J (2017) Impacts of Fish Tourism. In: Blumstein DT, Geffroy B, Samia DSM, Bess E (eds) Ecotourism’s Promise and Peril: A Biological Evaluation. Springer International Publishing, Cham, Switzerland, pp 59–72

Bilotta GS, Brazier RE (2008) Understanding the influence of suspended solids on water quality and aquatic biota. Water Res 42:2849–2861. https://doi.org/10.1016/j.watres.2008.03.018

Bourne CM, Kehler DG, Wiersma YF, Cote D (2011) Barriers to fish passage and barriers to fish passage assessments: The impact of assessment methods and assumptions on barrier identification and quantification of watershed connectivity. Aquat Ecol 45:389–403. https://doi.org/10.1007/s10452-011-9362-z

Bricknell IR, Bron JE, Bowden TJ (2006) Diseases of gadoid fish in cultivation: A review. ICES J Mar Sci 63:253–266. https://doi.org/10.1016/j.icesjms.2005.10.009

Brown CJM, Knight BW, McMaster ME, et al (2011) The effects of tertiary treated municipal wastewater on fish communities of a small river tributary in Southern Ontario, Canada. Environ Pollut 159:1923–1931. https://doi.org/10.1016/j.envpol.2011.03.014

Bunn SE, Arthington AH (2002) Basic principles and ecological consequences of altered flow regimes for aquatic biodiversity. Environ Manage 30:492–507. https://doi.org/10.1007/s00267-002-2737-0

Burke E (2016) Why Use Zebrafish to Study Human Diseases? NIH Intramural Research Program. https://irp.nih.gov/blog/post/2016/08/why-use-zebrafish-to-study-human-diseases. Accessed 24 Jul 2020

Callaghan DT, Blanchfield PJ, Cott PA (2016) Lake Trout (*Salvelinus namaycush*) spawning habitat in a northern lake: The role of wind and physical characteristics on habitat quality. J Great Lakes Res 42:299–307. https://doi.org/10.1016/j.jglr.2015.07.001

Canadian Council of Ministers of the Environment (1999) Canadian Water Quality Guidelines for the Protection of Aquatic Life: Dissolved Oxygen (Freshwater). In: Canadian Environmental Quality Guidelines. Winnipeg, Canada

Cano-Rocabayera O, Vargas-Amengual S, Aranda C, et al (2020) Mosquito larvae consumption in turbid waters: The role of the type of turbidity and the larval stage in native and invasive fish. Hydrobiologia 847:1371–1381. https://doi.org/10.1007/s10750-020-04195-0

Carlén O, Bostedt G, Brännlund R, Persson L (2021) The value of recreational fishing in Sweden – Estimates based on a nationwide survey. Fish Manag Ecol fme.12484. https://doi.org/10.1111/fme.12484

Chase DA, Flynn EE, Todgham AE (2016) Survival, growth and stress response of juvenile tidewater goby, *Eucyclogobius newberryi*, to interspecific competition for food. Conserv Physiol 4:cow013. https://doi.org/10.1093/conphys/cow013

Chauvin L (2018) The Unlikely Treasure off Peru’s Coast. Washington Post

Clark K, Pender D, Peterson MN, et al (2020) Reaching underserved populations through a fisheries education program. Fisheries 45:131–137. https://doi.org/10.1002/fsh.10390

Colwell RR (2002) Fulfilling the promise of biotechnology. Biotechnol Adv 20:215–228. https://doi.org/10.1016/S0734-9750(02)00011-3

Côté CL, Gagnaire PA, Bourret V, et al (2013) Population genetics of the American eel (*Anguilla rostrata*): FST = 0 and North Atlantic Oscillation effects on demographic fluctuations of a panmictic species. Mol Ecol 22:1763–1776. https://doi.org/10.1111/mec.12142

Currie S, Evans DH (eds) (2021) The Physiology of Fishes, 5th edn. CRC Press, Boca Rotan, Florida

Daily GC (1997) Nature’s Services: Societal Dependence On Natural Ecosystems. Island Press, Washington, United States

Dhillon RS, Fox MG (2004) Growth-independent effects of temperature on age and size at maturity in Japanese Medaka (Oryzias latipes). Copeia 2004:37–45. https://doi.org/10.1643/CI-02-098R1

Eliason EJ, Clark TD, Hague MJ, et al (2011) Differences in thermal tolerance among Sockeye Salmon populations. Science (80- ) 332:109–112. https://doi.org/10.1126/science.1199158

Evans DH, Piermarini PM, Choe KP (2005) The multifunctional fish gill: Dominant site of gas exchange, osmoregulation, acid-base regulation, and excretion of nitrogenous waste. Physiol Rev 85:97–177. https://doi.org/10.1152/physrev.00050.2003

Food and Agricultural Organization of the United Nations (2020) The State of the World Fisheries and Aquaculture 2020. Sustainability in Action, Rome

Food and Agricultural Organization of the United Nations (2012) Recreational Fisheries. Rome, Italy

Frankham R (1995) Effective population size/adult population size ratios in wildlife: A review. Genet Res 66:95–107. https://doi.org/10.1017/S0016672300034455

Frankham R, Ballou JD, Briscoe DA (2002) An Introduction to Conservation Genetics. Cambridge University Press, Cambridge, United Kingdom

Fréon P, Sueiro JC, Iriarte F, et al (2014) Harvesting for food versus feed: A review of Peruvian fisheries in a global context. Rev Fish Biol Fish 24:381–398. https://doi.org/10.1007/s11160-013-9336-4

Gibbons WN, Munkittrick KR (1994) A sentinel monitoring framework for identifying fish population responses to industrial discharges. J Aquat Ecosyst Heal 3:227–237. https://doi.org/10.1007/BF00043244

Golovanov VK (2006) The ecological and evolutionary aspects of thermoregulation behavior on fish. J Ichthyol 46:S180–S187. https://doi.org/10.1134/S0032945206110075

Gouskov A, Reyes M, Wirthner-Bitterlin L, Vorburger C (2016) Fish population genetic structure shaped by hydroelectric power plants in the upper Rhine catchment. Evol Appl 9:394–408. https://doi.org/10.1111/eva.12339

Government of Canada (2016) Mercury. https://www.canada.ca/en/health-canada/services/food-nutrition/food-safety/chemical-contaminants/environmental-contaminants/mercury.html. Accessed 18 May 2021

Hewitt LM, Servos MR (2001) An overview of substances present in Canadian aquatic environments associated with endocrine disruption. Water Qual Res J Canada 36:191–213. https://doi.org/10.2166/wqrj.2001.012

Holmlund CM, Hammer M (1999) Ecosystem services generated by fish populations. Ecol Econ 29:253–268. https://doi.org/10.1016/S0921-8009(99)00015-4

Hwang PP, Lee TH, Lin LY (2011) Ion regulation in fish gills: Recent progress in the cellular and molecular mechanisms. Am J Physiol - Regul Integr Comp Physiol 301:R28–R47. https://doi.org/10.1152/ajpregu.00047.2011

Jaeger KL, Olden JD, Pelland NA, Grimm NB (2014) Climate change poised to threaten hydrologic connectivity and endemic fishes in dryland streams. Proc Natl Acad Sci U S A 111:13894–13899. https://doi.org/10.1073/pnas.1320890111

Jellyman PG, Booker DJ, McIntosh AR (2013) Quantifying the direct and indirect effects of flow-related disturbance on stream fish assemblages. Freshw Biol 58:2614–2631. https://doi.org/10.1111/fwb.12238

Kelly PT, González MJ, Renwick WH, Vanni MJ (2018) Increased light availability and nutrient cycling by fish provide resilience against reversing eutrophication in an agriculturally impacted reservoir. Limnol Oceanogr 63:2647–2660. https://doi.org/10.1002/lno.10966

Kilgour BW, Munkittrick KR, Portt CB, et al (2005) Biological criteria for municipal wastewater effluent monitoring programs. Water Qual Res J Canada 40:374–387. https://doi.org/10.2166/wqrj.2005.041

Kjelland ME, Woodley CM, Swannack TM, Smith DL (2015) A review of the potential effects of suspended sediment on fishes: potential dredging-related physiological, behavioral, and transgenerational implications. Environ Syst Decis 35:334–350. https://doi.org/10.1007/s10669-015-9557-2

Kuhnlein HV, Humphries MM (2017) Traditional Animal Foods of Indigenous Peoples of Northern North America. In: Cent. Indig. Peoples’ Nutr. Environ. McGill Univ. Montr. http://traditionalanimalfoods.org/. Accessed 24 Jul 2020

Ledford JJ, Kelly AM (2006) A comparison of Black Carp, Redear Sunfish, and Blue Catfish as biological controls of snail populations. N Am J Aquac 68:339–347. https://doi.org/10.1577/a05-062.1

Lowe ML, Morrison MA, Taylor RB (2015) Harmful effects of sediment-induced turbidity on juvenile fish in estuaries. Mar Ecol Prog Ser 539:241–254. https://doi.org/10.3354/meps11496

Macnaughton CJ, Senay C, Dolinsek I, et al (2016) Using fish guilds to assess community responses to temperature and flow regimes in unregulated and regulated Canadian rivers. Freshw Biol 61:1759–1772. https://doi.org/10.1111/fwb.12815

Magoulick DD, Kobza RM (2003) The role of refugia for fishes during drought: A review and synthesis. Freshw Biol 48:1186–1198. https://doi.org/10.1046/j.1365-2427.2003.01089.x

McGinnis DF, Wuest A (2005) Lake Hydrodynamics. In: McGraw-Hill Yearbook of Science & Technology. McGraw-Hill Professional, New York, United States

Miles RD, Chapman FA (2006) The Benefits of Fish Meal in Aquaculture Diets. Fisheries and Aquatic Sciences Department, University of Florida, IFAS Extension. FA122

Modesto V, Ilarri M, Souza AT, et al (2018) Fish and mussels: Importance of fish for freshwater mussel conservation. Fish Fish 19:244–259. https://doi.org/10.1111/faf.12252

Mogdans J (2019) Sensory ecology of the fish lateral‐line system: Morphological and physiological adaptations for the perception of hydrodynamic stimuli. J Fish Biol 95:53–72. https://doi.org/10.1111/jfb.13966

Munkittrick KR, Dixon DG (1989) A holistic approach to ecosystem health assessment using fish population characteristics. Hydrobiologia 188/189:123–135. https://doi.org/10.1007/BF00027777

Munkittrick KR, Portt CB, Van Der Kraak GJ, et al (1991) Impact of bleached kraft mill effluent on population characteristics, liver MFO activity, and serum steroid levels of a Lake Superior White Sucker (*Catostomus commersoni*) population. Can J Fish Aquat Sci 48:1371–1380. https://doi.org/10.1139/f91-164

Neuheimer AB, Grønkjær P (2012) Climate effects on size-at-age: Growth in warming waters compensates for earlier maturity in an exploited marine fish. Glob Chang Biol 18:1812–1822. https://doi.org/10.1111/j.1365-2486.2012.02673.x

Noah L (2013) Whatever happened to the “Frankenfish”?: The FDA’s foot-dragging on transgenic salmon. Maine Law Rev 65:232–251

Noorhosseini SA, Lakani FB (2013) Ecological and biological effects of fish farming in rice fields. Persian Gulf Crop Prot 2:1–7

Norberg-King TJ, Embry MR, Belanger SE, et al (2018) An international perspective on the tools and concepts for effluent toxicity assessments in the context of animal alternatives: Reduction in vertebrate use. Environ Toxicol Chem 37:2745–2757. https://doi.org/10.1002/etc.4259

Patrick CJ, Yuan LL (2017) Modeled hydrologic metrics show links between hydrology and the functional composition of stream assemblages. Ecol Appl 27:1605–1617. https://doi.org/10.1002/eap.1554

Poff NL, Allan JD, Bain MB, et al (1997) The natural flow regime: A paradigm for river conservation and restoration. Bioscience 47:769–784. https://doi.org/10.2307/1313099

Ríos-Pulgarín MI, Barletta M, Mancera-Rodríguez NJ (2016) The role of the hydrological cycle on the distribution patterns of fish assemblages in an Andean stream. J Fish Biol 89:102–130. https://doi.org/10.1111/jfb.12757

Robinson CS, Tetreault GR, McMaster ME, Servos MR (2016) Impacts of a tertiary treated municipal wastewater effluent on the carbon and nitrogen stable isotope signatures of two darter species (*Etheostoma blennioides* and *E. caeruleum*) in a small receiving environment. Ecol Indic 60:594–602. https://doi.org/10.1016/j.ecolind.2015.06.041

Sánchez E, Colmenarejo MF, Vicente J, et al (2007) Use of the water quality index and dissolved oxygen deficit as simple indicators of watersheds pollution. Ecol Indic 7:315–328. https://doi.org/10.1016/j.ecolind.2006.02.005

Sandlund OT, Haugerud E, Rognerud S, Borgstrøm R (2013) Arctic Charr (*Salvelinus alpinus*) squeezed in a complex fish community dominated by perch (*Perca fluviatilis*). Fauna Nor 33:1–11. https://doi.org/10.5324/fn.v33i0.1579

Schiemer F (2000) Fish as indicators for the assessment of the ecological integrity of large rivers. Hydrobiologia 422/423:271–278. https://doi.org/10.1023/A:1017086703551

Schindler DE, Carpenter SR, Cole JJ, et al (1997) Influence of food web structure on carbon exchange between lakes and the atmosphere. Science (80- ) 277:248–251. https://doi.org/10.1126/science.277.5323.248

Schindler DW (2006) Recent advances in the understanding and management of eutrophication. Limnol Oceanogr 51:356–363. https://doi.org/10.4319/lo.2006.51.1_part_2.0356

Sternecker K, Geist J (2010) The effects of stream substratum composition on the emergence of salmonid fry. Ecol Freshw Fish 19:537–544. https://doi.org/10.1111/j.1600-0633.2010.00432.x

Stoll S, Fischer P, Klahold P, et al (2008) Effects of water depth and hydrodynamics on the growth and distribution of juvenile cyprinids in the littoral zone of a large pre-alpine lake. J Fish Biol 72:1001–1022. https://doi.org/10.1111/j.1095-8649.2007.01780.x

Suter GW, Barron M (2016) Generic Ecological Assessment Endpoints (GEAEs) For Ecological Risk Assessment: Second Edition With Generic Ecosystem Services Endpoints Added. US Environmental Protection Agency, Washington, United States

Tierney KB, Baldwin DH, Hara TJ, et al (2010) Olfactory toxicity in fishes. Aquat Toxicol 96:2–26. https://doi.org/10.1016/j.aquatox.2009.09.019

Torralva MDM, Angeles Puig M, Fernandez-delgado C (1997) Effect of river regulation on the life-history patterns of *Barbus sclateri* in the Segura river basin (south-east Spain). J Fish Biol 51:300–311. https://doi.org/10.1111/j.1095-8649.1997.tb01667.x

Tribot A-S, Carabeux Q, Deter J, et al (2018) Confronting species aesthetics with ecological functions in coral reef fish. Sci Rep 8:11733. https://doi.org/10.1038/s41598-018-29637-7

van der Oost R, Beyer J, Vermeulen NPE (2003) Fish bioaccumulation and biomarkers in environmental risk assessment: A review. Environ Toxicol Pharmacol 13:57–149. https://doi.org/10.1016/S1382-6689(02)00126-6

Vanni MJ (2002) Nutrient cycling by animals in freshwater ecosystems. Annu Rev Ecol Syst 33:341–370. https://doi.org/10.1146/annurev.ecolsys.33.010802.150519

Wegscheider B, Linnansaari T, Monk WA, Curry RA (2020) Linking fish assemblages to hydro‐morphological units in a large regulated river. Ecohydrology 13:e2233. https://doi.org/10.1002/eco.2233

Yang Z, Rannala B (2012) Molecular phylogenetics: Principles and practice. Nat Rev Genet 13:303–314. https://doi.org/10.1038/nrg3186

Yep B, Zheng Y (2019) Aquaponic trends and challenges – A review. J Clean Prod 228:1586–1599. https://doi.org/10.1016/j.jclepro.2019.04.290

Zasloff M, Adams AP, Beckerman B, et al (2011) Squalamine as a broad-spectrum systemic antiviral agent with therapeutic potential. Proc Natl Acad Sci U S A 108:15978–15983. https://doi.org/10.1073/pnas.1108558108
